# Supplementary material for: Panic in the Pandemic: Determinants of Vaccine Hesitancy and the Dilemma of Public Health Information Sharing during the COVID-19 Pandemic in Sri Lanka
Source: Int J Environ Res Public Health. 2024 Sep 24;21(10):1268. doi: 10.3390/ijerph21101268 (PMC11507827; doi:10.3390/ijerph21101268)
Supplement: Supplementary file 1 [file ijerph-21-01268-s001.zip › ijerph-3134644-supplementary.pdf]

Supplementary materials

1. Table S1: Socio-Demographic data of the study population

	Percentage	Number
**Household setting**		
Urban	64.29%	1964
Rural	19.48%	595
Estate	16.24%	496
**Age group**		
19 – 30	26.02%	795
31– 40	22.09%	675
41– 50	20.65%	631
51– 60	14.34%	438
> 60	16.89%	516
**Gender**		
Male	45.24%	1382
Female	54.76%	1673
**If Female, the marital, pregnancy or lactation status**		
Unmarried	15.77%	262
Married	80.07%	1330
Waiting to be pregnant	1.63%	27
Pregnant	3.55%	59
Lactating	8.25%	137
Neither pregnant nor lactating	1.99%	33
**If male, the marital status**		
Unmarried	25.22%	347
Married	74.49%	1025
Not willing to reveal	0.29%	4
**Ethnicity**		
Sinhala	83.2%	2482
Sri Lankan Tamil	10.83%	323
Indian Tamil	0.37%	11
Muslim	5.4%	161
Berger	0.07%	2
Other	0.13%	4
Not willing to reveal	0.0%	0
**Language proficiency- Understanding**		
Sinhala	92.1%	2873
Tamil	20.4%	637

English	22.8%	712

**Language proficiency- Speaking**		
Sinhala	89.9%	2884
Tamil	18.75%	584
English	12.3%	386
**Language proficiency- Writing**		
Sinhala	77.1%	2407
Tamil	16.8%	526
English	20.7%	648
**Religion**		
Buddhism	79.11%	2360
Islam	5.4%	161
Hindu	10.02%	299
Catholic	1.81%	54
Christian	3.49%	104
Other	0.17%	5
**Level of education**		
Not received any school education	4.86%	145
Primary [Grade 1-5]	16.43%	490
Secondary [Grade 6-11]	50.92%	1519
Tertiary [Grade 12-13]	19.14%	571
Higher education	8.65%	258
**Health status**		
On treatment for any illness	22.8%	680
Not on treatment for any illness	74.76%	2230
Not willing to reveal	2.45%	73
**Allergy status**		
Any previous food, drug or any other allergies	17.22%	511
No allergies	82.78%	2457
**Presence of Common NCDs**		
Diabetes mellitus	12.57%	373
Renal diseases	0.91%	27
Heart diseases	3.54%	105
Liver diseases	0.27%	8
Cancer/immune disorders	0.47%	14
Other diseases which need long term medication	2.06%	61
Long term steroid therapy	0.13%	4
Congenital disorders	0.37%	11
High blood pressure	12.57%	373

Other	9.47%	281
None	67.22%	1995
Not willing to reveal	2.63%	78

2. Survey Questionnaire

Question Number	Question
1.	Household setting, Under-privileged or Middle-income setting?
2.	Household setting, Urban, Rural, or Estate?
3.	Age group
4.	Gender
5.	Female, Marital, Pregnancy or Lactation status
6.	Male, Marital status
7.	Ethnicity
8.	Language Proficiency
9.	Religion
10.	Level of Education
11.	Health status
12.	Allergy status
13.	Chronic Non-communicable disease status
14.	What are the Vaccine types you know?
15.	Do you know about different technologies used in vaccine production?
16.	Do you know about differences in countries that produce vaccines?
17.	Do you know about the eligibility of different vaccines depending on age?
18.	Do you know about the effectiveness of Covid-19 vaccines?
19.	Do you know about the side-effects of Covid-19 vaccines?
20.	According to your knowledge, what is the purpose of Covid-19 vaccination?
21.	Do you make decisions on vaccination based on scientific evidence? If yes, what are your sources of information?
22.	Do you double-check the information in social media for accuracy? If yes, how?
23.	Are you aware of the vaccine eligibility criteria?
24.	Are you aware of the vaccine center locations?
25.	Are you aware of your vaccine eligibility status?
26.	Are you aware your vaccine eligibility period?
27.	How did you receive the information on vaccination process in your area?
28.	Level of satisfaction regarding the information received on Covid-19 vaccine
29.	What are the reasons for dissatisfaction on information received on Covid-19 vaccination?
30.	Did you receive Covid-19 vaccine, and if yes, the number of doses?
31.	What is the Covid-19 vaccine you received?
32.	Did any authorized officer requested your written or verbal consent before Covid-19 vaccination?
33.	Were you aware of the details in the consent form?

34.	Given the opportunity, would you prefer a different Covid-19 vaccine type?
35.	What is your first, second and third choices of Covid-19 vaccine type?
36.	How long it was taken to receive the vaccine once you're eligible to receive the Covid-19 vaccine?
37.	What are the reasons for the delay in receiving Covid-19 vaccine once eligible?
38.	What is the Covid-19 vaccine you received?
39.	What were the vaccine types you received if you had a different type of Covid-19 vaccine for the second and third doses?
40.	Were there any obstacles/delays in receiving the second dose? If yes, how long you had to delay the second dose?
41.	What are the reasons for the delay in receiving the second dose of Covid-19 vaccine?
42.	What are the side-effects of the Covid-19 vaccine that you were afraid of?
43.	How far did you had to travel to receive the Covid-19 vaccine?
44.	If you have not received ANY doses of Covid-19 vaccine, what are the reasons for not taking the vaccine?
45.	What are the side-effects you were afraid of if that was the reason?
46.	If provided with reliable information, would you take the Covid-19 vaccine?
47.	What do you suggest in improving Covid-19 vaccination program in the country?
48.	Do you think the Covid-19 vaccination should be a mandatory process? If yes, why? If no, why?
49.	If the vaccination certificate to be a mandatory document, what are the places it should be mandatory?
50.	Would you abide if the vaccination certificate was mandated to enter public spaces?

3. Knowledge of vaccines, vaccine manufacturing processes, effectiveness, side effects and vaccination program among participants

	Percentage	Number
**Aware of the Vaccine type**		
Oxford Astra Zeneca	31.36%	929
Sputnik V/Gamaleya	23.8%	705
Pfizer	73.53%	2178
Moderna	50.64%	1500
Sinopharm	88.22%	2613
Sinovac	6.45%	191
Johnson & Johnson	6.62%	196
Novavax	1.45%	43
Other	4.22%	125
**Aware of the different technologies used in vaccine production**		
Not at all aware	84.6%	2494
Very little informed	11.02%	325
Quite aware	3.09%	91
Moderately aware	1.19%	35
Extremely knowledgeable	0.1%	3
**Aware of the different countries in vaccine production**		
Not at all aware	35.89%	1058
Very little informed	39.21%	1156
Quite aware	17.84%	526
Moderately aware	5.36%	158
Extremely knowledgeable	1.7%	50
**Aware of the vaccine eligibility based on age**		
Not at all aware	66.52%	1961
Very little informed	18.25%	538
Quite aware	10.72%	316
Moderately aware	3.66%	108
Extremely knowledgeable	0.85%	25
**Aware of the vaccine efficacy**		
Not at all aware	55.39%	1633
Very little informed	22.46%	662
Quite aware	17.1%	504
Moderately aware	4.41%	130
Extremely knowledgeable	0.64%	19

**Aware of the vaccine side effects**		
Not at all aware	42.77%	1261
Very little informed	28.29%	834
Quite aware	17.88%	527
Moderately aware	8.82%	260
Extremely knowledgeable	2.24%	66
**The purpose of Covid-19 vaccination**		
Prevention of virus transmission	8.95%	263
Prevention of viral infection	10.76%	316
Disease prevention	14.5%	426
Minimize complications	35.14%	1032
Reducing the risk of dying	30.17%	886
Other	0.48%	14
**Aware of the vaccine eligibility criteria**		
Yes	77.41%	2262
no	22.59%	660
**Aware of the locations of the vaccination centers**		
Yes	96.1%	2808
no	3.9%	114
**Aware of your eligibility for vaccination**		
Yes	83.71%	2446
no	16.29%	476
**Aware of the date you become eligible for vaccination**		
Yes	80.84%	2362
No	19.16%	560

4. **Distribution of the use of scientific material in decision making, source of scientific information, and the awareness of the accuracy of the social media information**

	Percentage	Number
**Do you make decisions on vaccination based on scientific evidence**		
Yes	25.09%	737
No	37.96%	1115
Not known	36.94%	1085
**Sources of information**		
Social media	72.96%	537
Television / Radio programs	85.05%	626
Scientific publications	22.69%	167
Posters/pamphlets/etc.	20.38%	150
Scientific conferences, presentations	20.24%	149
Other	6.52%	48
**Double-check the information in social media for accuracy**		
Yes	27.91%	818
No	33.74%	989
Not known	38.35%	1124
**Methods of cross checking the information**		
Browsing internet articles	48.53%	397
Checking YouTube videos made by Sri Lankans	44.25%	362
Checking YouTube videos by foreigners	17.11%	140
Asking friends	73.23%	599
Asking the family doctor	33.62%	275
Other	5.87%	48
**How did you find out about the vaccination process in your area**		
Television/ Radio Channels	17.59%	513
Social media	18.55%	541
Posters	7.44%	217
Announcements	73.19%	2135
Through personnel	79.12%	2308
Other	7.1%	207

5. **Distribution of the level of satisfaction and reasons for dissatisfaction regarding information sharing**

	Percentage	Number
**Level of satisfaction with the information received regarding the COVID-19 vaccine**		
Very low	4.35%	127
Less	19.44%	567
Moderate	48.58%	1417
High	20.98%	612
Very high	6.65%	194
**Reasons for dissatisfaction with vaccine-related information**		
Lack of trust in authorities	31.94%	221
Delay in receiving information	42.63%	295
Difficulty understanding the content of medical information	53.32%	369
Confusion related to directions	23.12%	160
Language problems	16.62%	115
Not enough information	57.8%	400
Other	8.09%	56

6. **Distribution of vaccine recipients' details, vaccine choices, delays in vaccine receiving, reasons for vaccine delaying and refusal**

	Percentage	Number
**Type of the vaccine received**		
Sinopharm	60.27%	132
Sputnik V/Gamaleya	0.0%	0
Oxford Astra Zeneca	0.46%	1
Moderna	1.37%	3
Pfizer	25.57%	56
Other	0.0%	0
Not known	12.33%	27
**Number of the doses taken**		
One dose only	7.53%	219
Two doses	55.25%	944
Three doses [Up to first booster dose]	32.47%	1606
None	4.75%	138
**Consent in writing or verbally before receiving the vaccine**		
Yes	28.31%	62
No	50.23%	110
Not known	21.46%	47
**Awareness of the details contained in the consent form**		
**Yes**	61.29%	38
**No**	38.71%	24
**Given a choice of vaccine, Getting a different vaccine type**		
Yes	12.33%	27
No	42.47%	93
Not known	45.21%	99
**Preferred vaccine type- first choice**		
Sinopharm	7.41%	2
Sputnik V/Gamaleya	0.0%	0
Oxford Astra Zeneca	3.7%	1
Moderna	14.81%	4
Pfizer	70.37%	19
Other	3.7%	1
Not known	0.0%	0

**Preferred vaccine type- second choice**		
Sinopharm	3.7%	1
Sputnik V/Gamaleya	3.7%	1
Oxford Astra Zeneca	14.81%	4
Moderna	7.41%	2
Pfizer	51.85%	14
Other	0.0%	0
Not known	18.52%	5
**Preferred vaccine type- third choice**		
Sinopharm	0.0%	0
Sputnik V/Gamaleya	7.41%	2
Oxford Astra Zeneca	14.81%	4
Moderna	7.41%	2
Pfizer	44.44%	12
Other	3.7%	1
Not known	22.22%	6
**Duration taken to get the first dose of vaccine after receiving the invitation from the health officials**		
Less than a week	44.75%	98
Between a week and a month	40.64%	89
One month and 2 months period	12.79%	28
More than 2 months	1.83%	4
**Reasons for delaying vaccination after eligibility**		
Lack of knowledge on vaccination	4.27%	9
Fear of side effects	46.29%	123
Having strong faith in indigenous medicine	3.95%	21
Investigating the efficacy of vaccines	17.40%	96
Advising someone not to get the COVID-19 vaccine [eg; parents, community leader]	4.44%	60
Negative information about vaccines [eg; Use of vaccines to control humans through 5G technology]	12.46%	27
Absence of vaccination centers near the residence	3.69%	12
Lack of vaccines	3.06%	36
Lack of encouragement from relevant authorities	2.37%	24
Other	1.67%	24
**Delay in receiving the second dose**		
Received on the due date	66.07%	1669
Less than a month	22.09%	558
For a period of 1-2 months	9.11%	230
For a period of 2-4 months	2.53%	64
More than 4 months	0.2%	5

**Reasons for delaying the second dose**		
Vaccination centers have changed	11.37%	34
Lack of vaccines	38.13%	114
Misinformation	27.76%	83
Fearing and misinterpreting the side effects of the first dose	24.75%	74
Other	28.76%	86
**Distance needed to travel to get the vaccine**		
< 1 km	23.37%	640
2-5 km.	49.6%	1358
6 – 15 km.	18.81%	515
15 - 30 km.	1.42%	39
> 30 km.	0.69%	19
At a military headquarters or public vaccination center in another district	5.51%	151
In another country	0.29%	8
**Most concerned side effects of the Covid-19 vaccine**		
Fatigue	30.02%	822
Fertility problems	15.08%	413
Allergies	27.1%	742
Organ failure	37.69%	1032
Other	4.82%	132
No fear of side effects	41.64%	1140
**Reasons for vaccine refusal**		
Not yet qualified	1.46%	2
Fear of side effects	58.39%	80
Lack of opportunity to choose the type of vaccine required	7.3%	10
The vaccination process being a poorly organized and cumbersome process	1.46%	2
Reluctance to stand in long queues	7.3%	10
Having strong faith in indigenous medicine [e.g., herbal medicine]	17.52%	24
There is still no satisfaction about the efficacy of vaccines	43.07%	59
Advising someone not to get the COVID-19 vaccine [e.g., parents, community leader]	24.82%	34
Negative information about vaccines [e.g., Use of vaccines to control humans through 5G technology]	11.68%	16
Absence of vaccination centers near the residential area	0.0%	0
Other	18.25%	25
**Would you get the COVID-19 vaccine if you were well informed and comfortable with it?**		
Yes	62.04%	85
No	37.96%	52
